# Detection of Lesions Underlying Intractable Epilepsy on T1-Weighted MRI as an Outlier Detection Problem

**DOI:** 10.1371/journal.pone.0161498

**Published:** 2016-09-07

**Authors:** Meriem El Azami, Alexander Hammers, Julien Jung, Nicolas Costes, Romain Bouet, Carole Lartizien

**Affiliations:** 1 Université de Lyon, CREATIS; CNRS UMR5220; INSERM U1206; INSA-Lyon; Univ. Lyon 1, France; 2 Neurodis Foundation, Lyon, France; 3 PET Centre, Division of Imaging Sciences and Biomedical Engineering, King’s College London, London, United Kingdom; 4 INSERM U1028/CNRS UMR5292, Lyon Neuroscience Research Center, Lyon, France; 5 CERMEP-Imagerie du Vivant, Lyon, France; Chinese Academy of Sciences, CHINA

## Abstract

Pattern recognition methods, such as computer aided diagnosis (CAD) systems, can help clinicians in their diagnosis by marking abnormal regions in an image. We propose a machine learning system based on a one-class support vector machine (OC-SVM) classifier for the detection of abnormalities in magnetic resonance images (MRI) applied to patients with intractable epilepsy. The system learns the features associated with healthy control subjects, allowing a voxelwise assessment of the deviation of a test subject pattern from the learned patterns. While any number of various features can be chosen and learned, here we focus on two texture parameters capturing image patterns associated with epileptogenic lesions on T1-weighted brain MRI e.g. heterotopia and blurred junction between the grey and white matter. The CAD output consists of patient specific 3D maps locating clusters of suspicious voxels ranked by size and degree of deviation from control patterns. System performance was evaluated using realistic simulations of challenging detection tasks as well as clinical data of 77 healthy control subjects and of eleven patients (13 lesions). It was compared to that of a mass univariate statistical parametric mapping (SPM) single subject analysis based on the same set of features. For all simulations, OC-SVM yielded significantly higher values of the area under the ROC curve (AUC) and higher sensitivity at low false positive rate. For the clinical data, both OC-SVM and SPM successfully detected 100% of the lesions in the MRI positive cases (3/13). For the MRI negative cases (10/13), OC-SVM detected 7/10 lesions and SPM analysis detected 5/10 lesions. In all experiments, OC-SVM produced fewer false positive detections than SPM. OC-SVM may be a versatile system for unbiased lesion detection.

## Introduction

**Fig 1:**
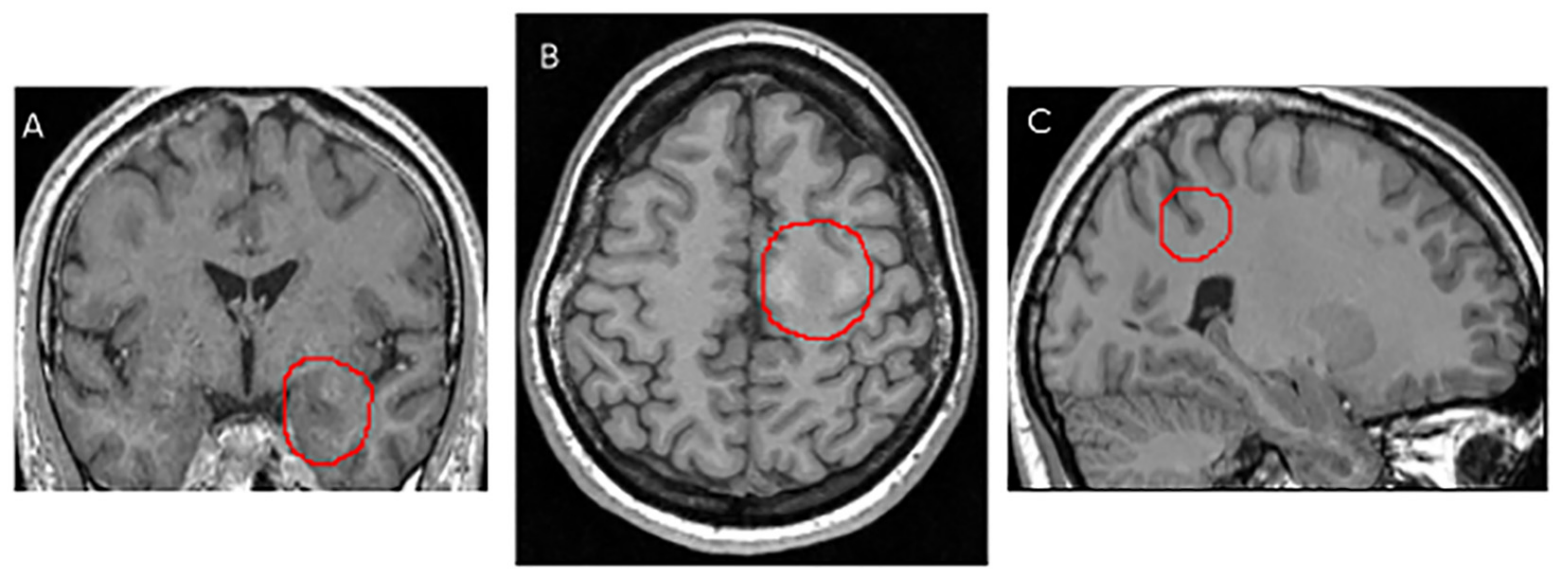
Example of three different epileptogenic lesions highlighted in red. A: (patient #4) coronal slice showing a hippocampus anomaly, B: (patient #1) axial slice showing signal and texture change, and C: (patient #2) sagittal slice showing a deep sulcus.

Malformative brain lesions occurring during cortical development are regularly associated with pharmacoresistant focal epilepsy. In particular, focal cortical dysplasias (FCDs) [1] are highly epileptogenic lesions that are often associated with intractable epilepsy and are present in up to 25% of patients with focal epilepsy. FCDs are mostly cortical abnormalities; histological subtypes I-III are distinguished [2]. If the neuronal migration from the subventricular zone to the cortex is disturbed during brain development, other malformations occur. For example, focal subcortical heterotopia are characterized by the presence of neurons (i.e. grey matter (GM)) located deep in the white matter (WM); there are also band-shaped heterotopia, and subependymal heterotopia next to the ventricles. The epileptogenic zone is usually centred on the dysplastic cortex. Its surgical removal often leads to freedom from seizures [3, 4]. Stereo-electro-encephalography (SEEG) or other forms of intracranial EEG are standard methods for identifying the epileptogenic zone. Less invasive methods based on the analysis of neuroimaging data increasingly help localize epileptogenic lesions. The pre-surgical planning is in general based on the analysis of surface video-EEG (VEEG) recordings, magnetic resonance imaging (MRI) with a special focus on T1-weighted and FLAIR sequences [5], positron emission tomography (PET) [6], and others. On MRI, the mainstay of imaging in pharmacoresistant epilepsy, FCDs may appear on T1-weighted images as cortical thickening, abnormally deep sulci, and blurring of the GM/WM interface, and may be associated with abnormalities of gyration [7, 8]. FLAIR hypersignal is also often present. The detection of malformative lesions is complex and challenging as they are very heterogeneous in terms of type, size and location (see examples in Fig 1). In the case of subtle changes, the lesions are easily missed by standard visual inspection of the image. Recent retrospective studies based on surgical epilepsy patients indicate that up to 33% with typical FCD type II lesions and 87% with FCD type I (i.e. intracortical) lesions present with unremarkable routine MRI [9]. Similarly, subtle heterotopia may only become apparent after MRI post-processing [10].

Computer aided diagnosis systems (CAD) based on MRI have been proposed in the past few years to assist in the screening of epileptogenic lesions. Among them, one can distinguish between patient classification methods [11–14] and voxelwise classification techniques [15–21]. In the first group, the diagnosis is made at the patient level and the goal is either to discriminate patients from healthy controls, or to perform a lateralization, or rough localization of the epileptogenic lesion. In the more challenging voxelwise classification, the voxelwise classification into normal versus pathological usually results in a cluster map indicating the most suspicious regions. One way of doing so consists in performing a binary classification where the decision model is learned on a series of feature vectors selected from normal and pathological locations (voxels, vertices) on patient scans [20, 21]. The main difficulty with this approach is to accurately capture the characteristics of malformative lesions including FCD and heterotopia when very few pathological examples are available for a large number of possible locations. The scarcity of pathological examples is partly due to the difficulty of defining a gold standard on these examples. Annotated samples may not fully represent the physiopathological heterogeneity of epileptogenic lesions (as illustrated on Fig 1) as well as the image pattern variability that is likely to be observed for any lesion type depending on its location in the cortex or white matter. The alternate solution is to perform comparisons between a patient and a cohort of normal subjects [16, 18, 19]. In these studies of FCD mapping, discriminant features were extracted at the voxel level from structural MRI and fed into a mass univariate statistical analysis based on a general linear model (GLM). Features included relative MR signal intensity and texture parameters as well as distribution of the grey and white matter for voxel-based morphometry (VBM) [22]. These studies are most commonly performed within the framework of the statistical parametric mapping software (SPM: fil.ion.ucl.ac.uk/spm; Wellcome Trust Centre for Neuroimaging).

Here, we propose a novel flexible classification method combining textural maps [23] relevant for FCD and heterotopia associated abnormalities and a one-class support vector machine (OC-SVM) [24] that is trained with ‘negative’ (normal) examples from a control database only. The model then allows the detection of novelty (i.e. abnormalities) in the test group on a voxelwise basis. The OC-SVM method has been successfully applied in many application domains, a review of which may be found in [25], but rarely in the field of neuroimaging [26, 27]. The main contribution of this work is a voxel-level machine learning system that performs outlier detection in neuroimaging data based on multivariate features. Feature extraction is adapted to capture the specificity of malformative epileptogenic lesions including FCD and heterotopia. We hypothesize that the OC-SVM framework overcomes the main limitations of the mass univariate statistical analysis by allowing 1) to better control outliers within the learning step, 2) to easily incorporate multiple feature maps in a multivariate analysis without any assumption on the feature statistical distribution and 3) opening the way for the integration of spatial *a priori* information that enables learning from the voxel and its neighbourhood.

We had already investigated the potential of such a method in a preliminary study [28]. Here, we extend the architecture of the OC-SVM CAD system and complete its evaluation. First, we propose a novel method to normalize the outlier scores produced by the OC-SVM classification scheme, thus allowing correcting for the type I error. We now evaluate the CAD system on synthetic data and on a larger patient group, including a quantitative performance analysis against manually annotated epileptogenic lesions. Finally, we compare the OC-SVM scheme against the mass univariate SPM analysis optimised for this application. Our results indicate that the OC-SVM scheme outperforms the SPM analysis and competes favourably with more sophisticated systems based on a two-step image processing.

## Data description

### Study group

The study was approved by our institutional review board (Comité de protection des Personnes Sud-Est IV) with approval number: 140277A-12 and 2012-A00516-37 and written informed consent was obtained for all participants.

*Clinical patient group:* The clinical test group was composed of 11 patients admitted to Lyon’s Neurological Hospital for medically intractable epilepsy. Five of these patients were operated upon between 2009 and 2013 and found to have histologically proven FCD. The presurgical workup included standard MR neuroimaging as well as SEEG and VEEG. Some patients also had a PET and/or a MEG as part of their clinical workup when clinically indicated. Post-processing of these anonymised scan data acquired for clinical purposes did not require individual consent from the individuals who had been scanned. The second series included six patients who were admitted between 2014 and 2015 following the same inclusion criteria as those of the first group of five patients. These patients underwent a similar presurgical evaluation except that the MR imaging protocol was slightly different as explained in the next section. They also underwent systematic PET and MEG as part of a research protocol. All patients had an initial routine radiological assessment consisting in a blind visual inspection of standard T1-weighted 1.5 T MRI by two expert neurologists. Three FCD lesions were visually detected on three patients thus diagnosed as MRI-positive (MRI+). The same experts also reported the two hippocampal atrophies (HA) of patient #4 and patient #6. The two FCD lesions of the right amygdala for patient #4 and of the right temporal lobe for patient #6 were not visible on the T1-weighted MRI. These areas were suspicious on VEEG and SEEG respectively and lesions confirmed on histology. These two patients were thus classed as MRI-negative (MRI-). The MRI- group also included the six remaining patients.

*Normal subject database:* Two learning databases were used in this study to match the characteristics of the two series of clinical data detailed above. The first database referred to as NDB1 consisted of 37 T1-weighted MRI exams acquired on healthy control subjects aged 18-53 years. The second one referred to as NDB2 consisted of 40 T1-weighted MRI exams acquired on healthy control subjects aged 20-62 years. Both databases were visually inspected to exclude subjects with significant structural abnormalities.

*Simulated patient group:* Five additional MRIs of healthy control subjects (simulation subjects) acquired following the same protocol as for NDB1 were used to simulate two kinds of epileptogenic lesions.

### MRI acquisition

Subjects from NDB1, the simulated patient group and the first series of 5 patients all had a 3D anatomical T1-weighted brain MRI sequence (TR/TE 9.7/4 ms; 176 sagittal slices of 256 × 256 millimetric cubic voxels) on a 1.5 T Sonata scanner (Siemens Healthcare, Erlangen, Germany).

Subjects from NDB2 and the second series of 6 clinical cases had a 3D anatomical T1-weighted brain MRI sequence on the same scanner but with a slightly different protocol (TR/TE 2400/3.55; 160 sagittal slices of 192×192 1.2 mm cubic voxels).

## Description of the CAD system

**Fig 2:**
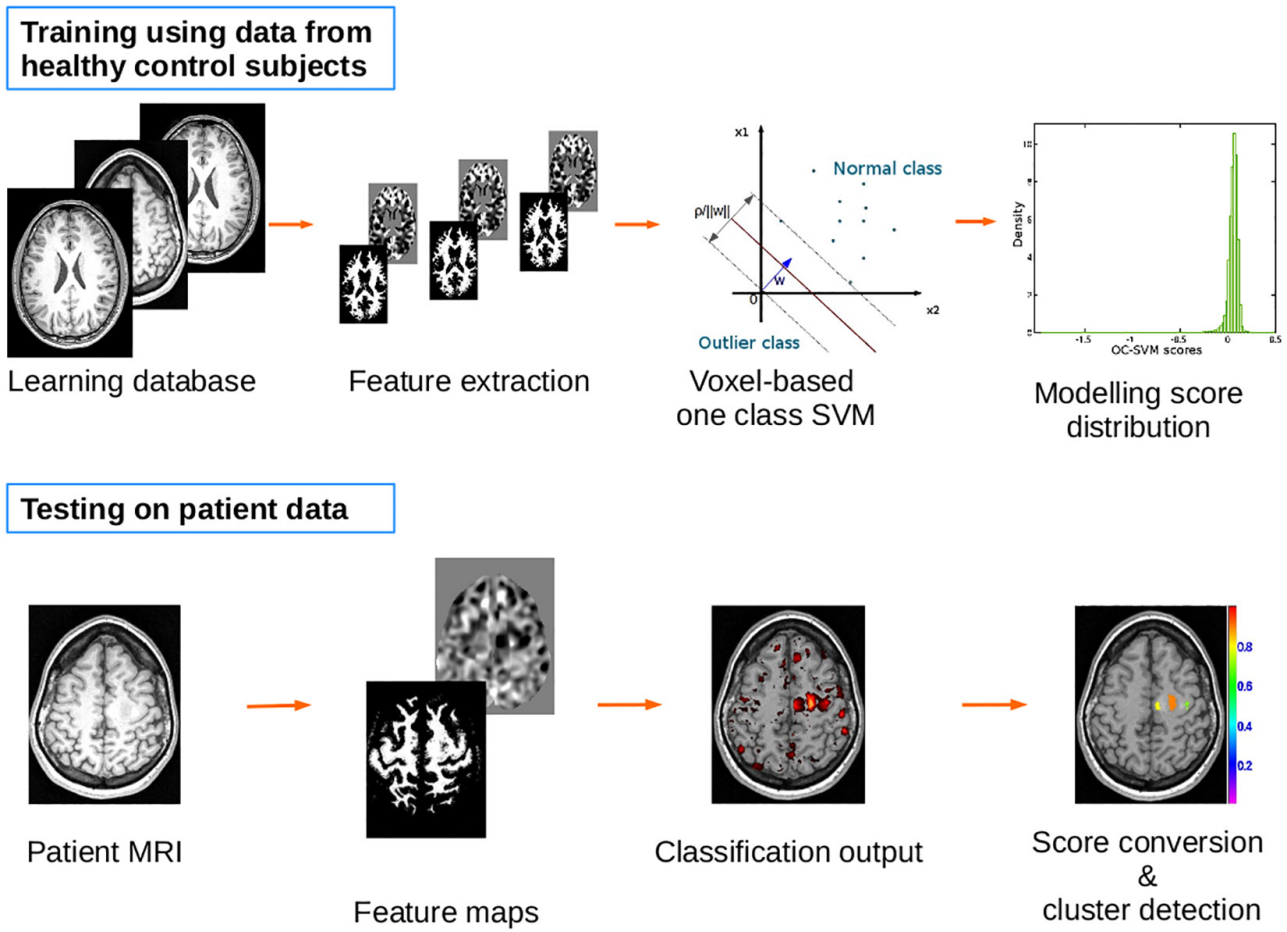
Scheme of the CAD system illustrating the learning (top) and the testing phase (bottom).

Fig 2 schematizes the different steps of the proposed CAD system. The steps are further described in the remainder of this section.

### Data preprocessing

Following the work in [23], the preprocessing was performed based on the reference methods implemented in SPM8. The spatial normalization was performed using the unified segmentation algorithm [29]. This method combines all steps including the segmentation of the different tissue types, namely grey matter (GM), white matter (WM) and cerebrospinal fluid (CSF), correction for magnetic field inhomogeneities and spatial normalization under the same objective function that is iteratively solved. The 3D brain MRI of each subject was normalized to the standard brain template of the Montreal Neurological Institute (MNI) [30] as contained in SPM8 using the default parameters for normalization and a voxel size of 1 × 1 × 1 mm as in [29]. The cerebellum and brain stem were excluded from the spatially normalized images to restrict the analysis to brain regions susceptible to harbour FCDs. The masking image in the reference MNI space was derived from the Hammersmith maximum probability atlas [31]. The resulting volume of interest contained 1.5 million voxels (≈ 1.5 liters).

### Feature extraction

Previous work [11, 15, 19, 23], referenced in the introduction, showed the discriminative power of different types of features for the detection of epileptogenic lesions on T1-weighted MRIs. This includes grey-level intensities of the T1-weighted images, volume of specific brain structures such as hippocampus, tissue class probabilities, image maps highlighting FCD characteristics, as well as second order texture features based on grey level co-occurrence matrices. In this study, we hypothesized that the CAD system would be selectively specific to FCD and heterotopia as typical epileptogenic lesions by considering only the features that model the clinical description of this type of lesions and that were previously shown to be discriminant (see for instance [23] and [32]). Two parametric maps were thus computed for all subjects from the probabilistic tissue maps to capture suspicious patterns characterizing 1) the extension of the GM into the WM, referred to as extension map and 2) the junction between the GM and WM, referred to as junction map.

The extension map was obtained from the segmented GM image derived from the segmentation by smoothing with a Gaussian kernel of width 6 mm. To compute the junction map, the T1-weighted intensity corrected MR image was transformed into a binary image by selecting voxels whose grey value ranges between low threshold $T_{low}=mean_{GM}+\frac{1}{2}SD_{GM}$ and high threshold $T_{high}=mean_{WM}-\frac{1}{2}SD_{WM}$ where *mean* and *SD* values correspond to the mean and standard deviation of the grey values in the respective tissue class, with *mean*_*WM*_ > *mean*_*GM*_. A smoothing with a 6 mm width Gaussian kernel was then applied to the binary image. Details concerning the computation of theses maps are given in [23].

**Fig 3:**
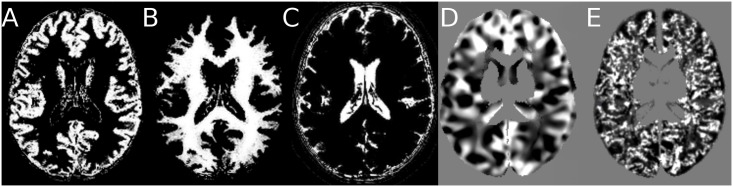
Example slice of: (A) GM probability map (B) WM probability map (C) CSF probability map (D) extension map (E) junction map.

For each feature type and for each healthy control database (NDB1 and NDB2), a mean parametric map (referred to as “mean template”) and a standard deviation map (referred to as “SD template”) were created by averaging and computing the standard deviation over the parametric maps computed from the 37 control subjects of NDB1 and from the 40 control subjects of NDB2, respectively. The final individual parametric map, also called Z-score map, was obtained by subtracting the mean parametric map (mean template) from the parametric value of the individual map and dividing by the standard deviation template. As a result, high signal indicates typical MRI features of epileptogenic lesions. Each voxel *k* from the volume of interest was thus described by a two component feature vector **V**_*k*_ comprising the extension and the junction values at this voxel location. Fig 3 gives example slices of the two feature maps as well as the corresponding individual tissue probability maps.

### OC-SVM classifier design

The OC-SVM approach [24] allows learning from negative examples only while keeping all the desirable properties of the support vector machines (SVM). This includes combining different maps by specifying the features to be included in the learning step (multivariate approach), using a kernel function to find non-linear decision boundaries, and yielding a sparse representation of this decision boundary.

#### OC-SVM methodology

The one-class SVM methodology proposed by Schölkopf et al. [24] is a special case of the SVM algorithm [33] for assigning labels *y*_*i*_ ∈ {−1, 1} corresponding to two distinct classes of objects, based on *n* training samples ${\left(x_{i},y_{i}\right)}_{i=1,\ldots ,n},\;x_{i}\in R^{p}$ from the negative class only. The learning samples are first mapped into a higher dimensional space via a feature map *ϕ* associated with a kernel *K* such as *K*(**x**_*i*_, **x**_*j*_) = (*ϕ*(**x**_*i*_).*ϕ*(**x**_*j*_)). This kernel is chosen to maximize the separability of the data from the origin of the feature space. An example is the radial basis function (RBF) kernel where: $K\left(x_{i},x_{j}\right)=\exp\left(-\frac{∥x_{i}-x_{j}{∥}^{2}}{2\sigma^{2}}\right).$

In the transformed space, the origin is treated as the only member of the negative class and the objective is, like in the two-class SVM case, to find the hyperplane **w**^⊤^**x**+*ρ* that separates the learning examples from the origin with maximum margin.

The associated minimization problem is:

$$\begin{array}{c} \left\{\begin{array}{ccc} \min_{w,\rho ,\xi_{i}} & \frac{1}{2}{∥w∥}^{2}+\frac{1}{\nu n}\sum_{i=1}^{n}\xi_{i}-\rho & \\ \text{subject}\;\text{to} & \left(\phi \left({\text{x}}_{i}\right).w\right)\geq \rho -\xi_{i},\; & i\in [1,n]\\ \text{and} & \xi_{i}\geq 0, & i\in [1,n], \end{array}\right. \end{array}$$ (1)

where *ξ*_*i*_ are slack variables which allow the relaxation of the inequality constraints to account for cases with nonseparable classes and $\frac{1}{\nu n}$ is the associated regularization parameter that controls the trade-off between model complexity and the number of errors. The parameter *ν* corresponds to an upper bound on the fraction of permitted outliers (the so-called *ν* − property).

For a voxel *k*, the output of the OC-SVM is the signed distance from the optimal hyperplane found during the learning phase at this voxel location. As we only learn from normal examples, most training examples will have a positive signed distance to the optimal separating hyperplane and only a fraction of the examples (controlled by *ν*) will have a negative signed distance.

#### Implementation

Each voxel *k* from the MRI scan was separately associated with a OC-SVM classifier. The classifier was trained using the matrix $M^{k}\in M_{n,p}\left(R\right)$ with *n* = 37 for NDB1 and *n* = 40 for NDB2 and *p* = 2 where each row of *M*^*k*^ is an instance of the feature vector **V**_*k*_. A RBF kernel was used and the values of *ν* and the kernel parameter *σ* were derived as described below. The 1.5 million OC-SVM predictive models were computed. These models were applied to test images to produce a OC-SVM distance map, with the same dimensions as the normalized input image, where each voxel value is the local OC-SVM distance to the local hyperplane found during the learning step. The Matlab toolbox developed by Canu et al. [34] was used to solve the optimization problem (Eq 1) at each voxel location.

#### Cluster identification and analysis

Thresholding the OC-SVM distance map allows identifying clusters of voxels that will be regarded as pathological. As outlined above, the more negative the score, the more suspicious (pathological) the voxel. Selection of the threshold thus controls the trade-off between the sensitivity and the specificity of the CAD system.

We developed a novel method to adjust the threshold value so as to control the type I error (false positive detection rate). The idea is to model the distribution of the OC-SVM scores for normal voxels and then infer the probability for any given test voxel to be abnormal considering its score value relative to the normative distribution. This normative score distribution was computed by performing a leave-one-out procedure on the normal subjects. For example, the first database (NDB1) comprised *n* = 37 healthy control subjects. For each voxel, a OC-SVM model was trained using feature vectors corresponding to *n* − 1 healthy control subjects, and tested on the remaining healthy control subject. This leave-one-out resulted in 37 OC-SVM score maps. The histogram of each OC-SVM score map was then computed by specifying the same range and bin intervals. The normative score distribution was obtained by averaging all 37 histograms. This distribution was then approximated by a non-parametric distribution using a kernel density estimator [35] to convert score values into probability density estimates.

**Fig 4:**
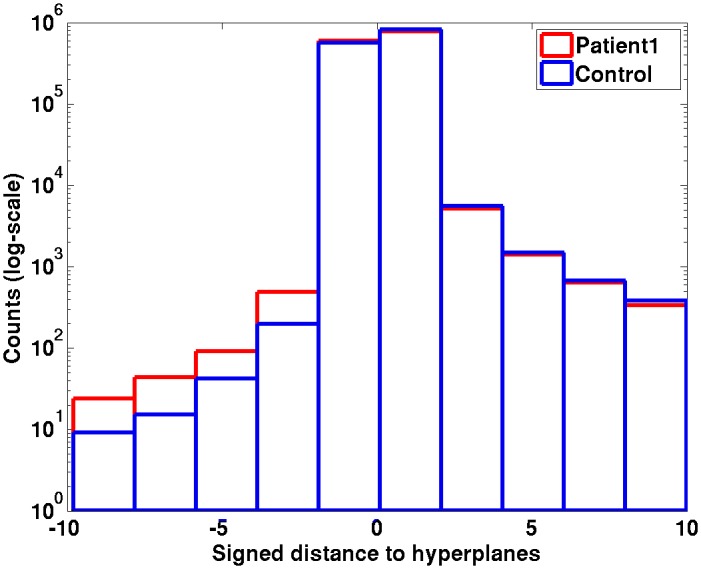
Example of OC-SVM score histogram (on a log-scale) obtained for a control subject from NDB1 (blue) overlaid with that of patient #1 (red). There are differences in a small number of voxels, all with a negative signed distance to the hyperplanes indicating non-normal tissue.

We assumed that the OC-SVM score distribution of any given test patient can be represented by this normative score distribution considering that it is not influenced by the small fraction of outlier examples (<1%) that are likely to correspond to typically sized lesions. This is equivalent to considering the OC-SVM scores for abnormal examples as outliers of the distribution of normal example scores. The type I error can then be controlled by using a threshold value that corresponds to a given p-value on the normative distribution. The good overlap between the score distribution of a control subject and that of a test patient in Fig 4 illustrates the validity of this hypothesis. We set a p-value of 0.001 which is equivalent to a signed distance of -0.95 for NDB1 and -1.07 for NDB2 given the two normative score distributions estimated in this study. The clustering process then consists in scanning the thresholded map in a lexicographical voxel order and aggregating all non-null voxels that are linked for the 26-connectivity rule. Descriptive statistics per cluster (size, minimum, maximum and mean of the voxel scores) are then computed. Each cluster is assigned a value that corresponds to the minimum OC-SVM score value of its constituting voxels, i.e. the smallest probability value of belonging to the normal class. Finally, a labelled cluster map is generated in which cluster order is given by the minimum (i.e. most pathological) OC-SVM score value.

### SPM analysis

The mass univariate single subject analysis [22] was performed within the framework of the statistical parametric mapping software (SPM: fil.ion.ucl.ac.uk/spm; Wellcome Trust Centre for Neuroimaging). A general linear model (GLM) is first fitted to each voxel based on user-predefined factors of interest (e.g. groups, frequency of seizure, etc) and/or confounding variables (e.g. sex, age) [22]. Then, post-hoc inferences on the effect of interest are made with a standard mass univariate statistical test resulting in statistical maps of T or F values for each factor of interest. The statistical significance of clusters of voxels that exceed an uncorrected statistical threshold in the SPM is evaluated within the Gaussian random fields (GRF) theory which allows correcting p-values for multiple testing in the search volume and correlation among neighbouring voxels (due to spatial smoothing). SPM also allows performing conjunction analysis as defined in [36, 37] to test the global null hypothesis that there is a conjunction of one or more effects, i. e. the factors of interests were consistently and jointly significant.

To compare each individual patient against the control group, we considered the same extracted feature maps, used as an input to the OC-SVM classifier, to perform a ‘one-way ANOVA’ based on the following four factors of interest: patient junction map, control junction maps, patient extension map, and control extension maps. We used two contrasts [1,-1,0,0] and [0,0,1,-1] to test for significant increases in the patient junction and extension maps compared to controls. A first analysis consisted in thresholding the two resulting T-score maps using a p-value of *p* = 0.001, to produce a cluster map where each cluster was characterized by its size and the maximum T-score value of its constituting voxels. Clusters with the highest T-scores were considered as most suspicious. A conjunction analysis of these two effects (junction and extension), as defined above, was also performed using the same p-value of 0.001.

## Evaluation of the CAD system

Performance evaluation of the two CAD systems, based on the OC-SVM and SPM formalism respectively, followed a two-step protocol. We first performed a quantitative analysis based on simulated T1-weighted MR images featuring realistic lesions. This allowed controlling the ground truth and computing metrics derived from a standard receiver operating curve (ROC) analysis. We then assessed the qualitative performance achieved on clinical data of FCD patients by comparing the lesions obtained by the CAD systems with those manually outlined by neurologists based on their expert reading. In deciding on the location of the epileptological zone, electroclinical information, i.e. seizure semiology and EEG features, as well as PET or MEG data when available, were taken into account.

### Evaluation on realistic simulation data

We simulated two types of typical epileptogenic abnormalities described in the introduction section, firstly a focally blurred junction between grey and white matter and secondly, heterotopion-like lesions resulting from the presence of GM in the white matter. Five MRI scans of healthy subjects from the simulation group were used to perform all simulations.

#### Simulation of blurred junctions

**Fig 5:**
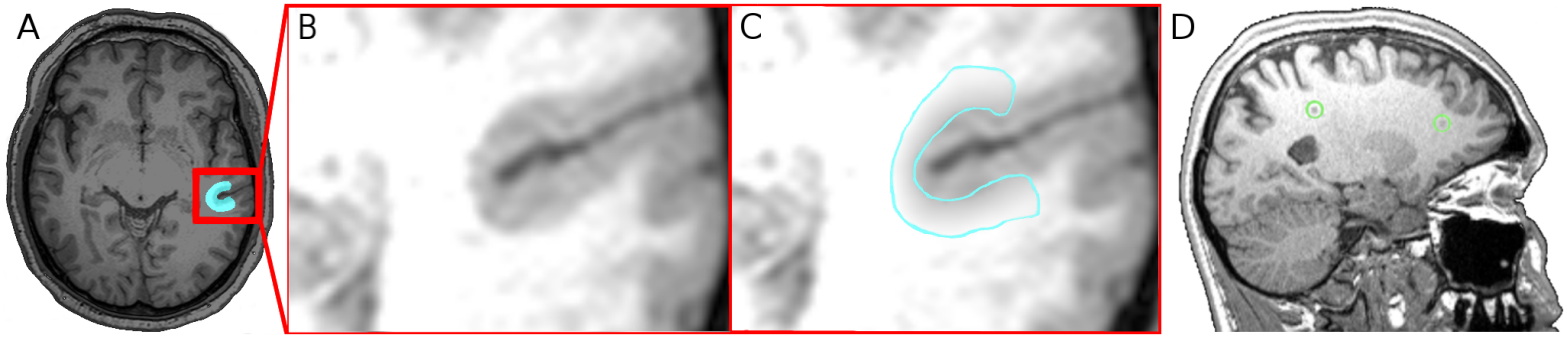
Realistic simulations. (A) Example slice of the original MRI where the alteration location is highlighted in blue, (B) zoom on the original MRI before introducing the alteration, (C) and zoom on the introduced junction alteration. The introduced lesion has a very low contrast and is almost impossible to detect with the naked eye. (D) Example of a simulation subject sagittal MRI slice showing two heterotopion-like lesions (within the green circles) that were simulated using GM values selected within the range *I* of grey-level values.

The simulation of subtle junction alterations included the following steps: **1-** in the native T1-weighted MRI of a simulation subject, 2D U-shaped regions of interest (ROI) around the grey matter at the bottom of a sulcus were drawn on six consecutive slices to capture 3D information (see Fig 5). **2-** the grey-level value histogram of the voxels within the junction ROI was computed. **3-** the voxels with grey-level values ranging in the GM/WM interface defined by $I=[mean_{GM}+\frac{1}{2}SD_{GM}\;,\;mean_{WM}-\frac{1}{2}SD_{WM}]$ (with *mean*_*WM*_ > *mean*_*GM*_) were isolated. **4-** a mean filter, a disk of radius 3 mm (≈ 28 voxels) was applied to the entire original MRI. **5-** In the original MRI, values of the voxels selected in step **3** and within the ROI were substituted by their corresponding values in the filtered image. This processing ensures that only the voxels within the GM/WM interface are altered, closely resembling epileptogenic FCDs with blurred junctions [23].

We simulated one lesion for each simulation subject, resulting in a total of 5 FCD-type junction lesions. Fig 5A, 5B and 5C show an example of a very subtle U-shaped lesion.

#### Simulation of heterotopion-like lesions

For each of the five simulation subjects, six heterotopion-like lesions were simulated at different locations in the WM. To control the inter-subject variability due to lesion location, these lesions were simulated at the same approximate location in all five subjects. A binary mask containing six spherical lesions of radius 2 mm was drawn in the MNI space. This mask was then mapped to each individual subject native space to set the location and size of the six simulated lesions. To simulate challenging detection cases of heterotopion-like lesions, grey-level values of the heterotopion-like lesions were selected by randomly sampling values within the same range $I=[mean_{GM}+\frac{1}{2}SD_{GM}\;,\;mean_{WM}-\frac{1}{2}SD_{WM}]$ that was used for the blurred junctions which is likely to be less contrasted and smaller than usual. The standard values (*mean and standard deviation*) for each tissue class (GM and WM) were extracted from the grey-level histogram of each individual subject’s MRI. A total of 5 (number of simulation subjects)×6 (number of different locations) = 30 heterotopia was simulated. Fig 5D gives an example slice of the heterotopion-like lesions for a simulation subject displayed in native space.

#### Comparison with the ground truth for synthetic data

A ROC analysis [38] was performed based on OC-SVM and SPM score maps (T-scores for SPM and distances to the hyperplane for OC-SVM). The ROC curve reports coupled values of the true positive rate (TPR) and false positive rate (FPR) for different values of the decision threshold. In this study, the ROC curve was computed at the voxel level, thus meaning that a voxel was recorded as a true positive if its score value exceeded the threshold and was located in one of the simulated lesions. This type of voxel analysis avoids defining mark-labelling rules that may bias the performance assessment [39]. For a test patient, the threshold was varied to cover the entire range of T-scores (SPM) or OC-SVM scores for a test patient so as to homogeneously sample the ROC curve. Performance was also summarized by the standard area under the ROC curve (AUC). Confidence intervals on the AUC estimates were computed by using bootstrap estimates at the patient level for both the SPM and OC-SVM methods. One hundred and twenty bootstrap samples were obtained by choosing with replacement among all simulated patients repeated for each anomaly. For each bootstrap sample, the score maps (SPM T-scores or OC-SVM scores) of the selected simulated patients were concatenated to form a global score vector that was used to compute one bootstrap AUC estimate. Confidence intervals for the differences in AUC estimates between the different CAD configurations were finally derived according to the bias-corrected percentile method described in [40].

For clinical applications with highly imbalanced datasets (in our case, lesions have a small size compared to the entire volume (<1%)), the TPR value for a fixed low value of the FPR should also be reported [41]. In this study, a FPR of 0.1 corresponds to the detection of 150 000 voxels (10% of the 1.5 million voxels) which is already more than 100 times the size of the true lesion. We thus chose to compare the performance at three fixed FPR values of 0.01, 0.05 and 0.1, respectively.

### Evaluation on clinical data

For clinical data, manual labelling is not available for all patients. Only three out of thirteen lesions (MRI positive cases) were manually delineated by an expert. In the remaining cases, the ground truth corresponds to a consensus of two experts who analysed all available neuroimaging data (e.g. PET, MEG and SEEG) in order to give an approximate localization of the epileptogenic zone. The clusters detected either by the OC-SVM or by the SPM analysis were compared with this ground truth for each of the eleven patients considered in this study. Clusters were designated by an expert as false-positive detections, or as identifying an abnormal region comprising the epileptogenic lesion. Considering the expected size of clusters given by the SPM analysis (82 voxels = 82 mm^3^), we only reported clusters that were superior to 82 voxels for the SPM and the OC-SVM map as both analyses were performed using the same input feature maps. Sensitivity was defined as the fraction of detected clusters that correctly colocalized with the expert report. In lieu of specificity, we report the mean number of false positive (FP) clusters per patient scan. Separate evaluations were performed for the 3 MRI+ and the 8 MRI- cases.

## Results

### OC-SVM parameter optimization

The OC-SVM algorithm with a RBF kernel has two hyper-parameters: the standard deviation of the RBF kernel *σ* and the parameter *ν*. Ideally, these hyper-parameters should be optimized at each voxel location. For each voxel, an estimate of the mean error rate can be obtained by performing a leave-one-out optimization procedure based on the 37 healthy control subjects of NDB1 and idependently on the 40 control subjects of NDB2. This procedure has however a prohibitively high computational cost. To reach a compromise between computational efficiency and detection performance, a subset of randomly chosen voxels can be used. To determine the minimum number of voxels required to obtain representative estimates of the mean error rate that would be obtained if all 1.5 million voxels were used, we computed the mean error rate (i.e. the FPR) for varying pairs of (*ν*, *σ*) for increasing numbers of voxels. Convergence is obtained from approximately 1000 voxels; adding more voxels do not change the value of the mean fraction of errors, and this convergence is very similar for different value pairs of the hyper-parameters. The mean error rate (i.e. the FPR) over 4000 randomly chosen voxels was considered as the optimization criterion. For this empirically determined number of 4000 voxels, we then selected a single global value pair (*ν*, *σ*) for the hyper-parameters used for all voxel locations.

The value of *ν* was varied in the interval [0.01…0.56] using eight intervals on a *log*_10_ scale. The value of *σ* was varied in the interval [2^−4^ …2^4^] using eight intervals on a *log*_2_ scale.

**Fig 6:**
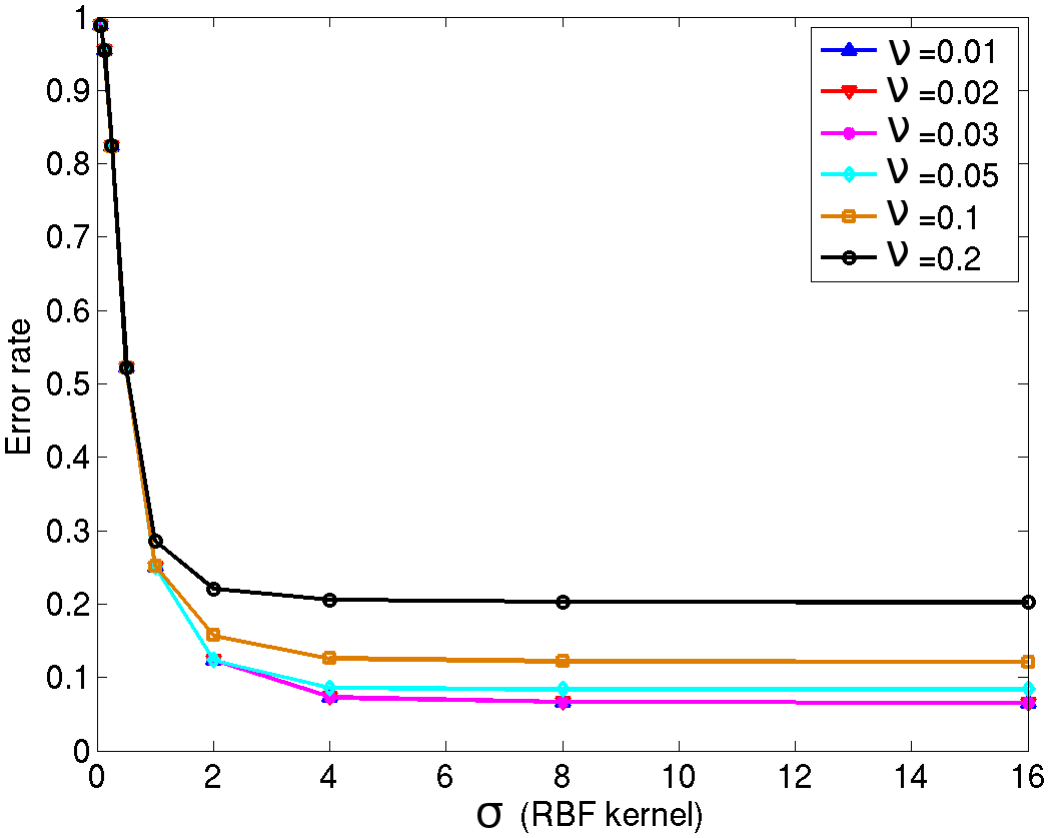
Hyper-parameter optimization curve. The bigger the width of the RBF kernel, the worse the generalisability due to the risk of under-fitting. Similarly, the smaller the value of *ν*, the higher the risk of over-fitting (fewer observations may be excluded); for better generalisability and given noise in medical images, the value should not be too small. Here, the pair (*ν* = 0.03, *σ* = 4) is therefore the optimal combination.

Fig 6 shows the resulting mean error rate curves for the different values of *ν* and *σ* for NDB1. Similar curves were obtained for NDB2. The values *ν* = 0.03 and *σ* = 4 for NDB1, *ν* = 0.05 and *σ* = 3 for NDB2 were shown to produce the smallest FPR while producing the least complex model (in terms of dimensionality and memory load).

### Comparison of OC-SVM and SPM detection performance

#### Simulated data results

**Fig 7:**
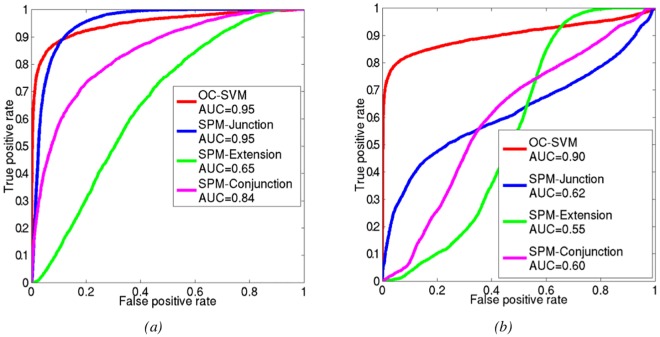
Comparison of OC-SVM and SPM performance for the simulated blurred junction and heterotopion-like lesions.

Fig 7a shows the ROC curves corresponding to the detection of the five blurred junctions. For these lesions, the OC-SVM approach and the SPM analysis based on the junction contrast perform equally in terms of AUC (AUC = 0.95) while the SPM conjunction analysis has an intermediate performance with AUC values of 0.84. As expected the SPM analysis based on the extension contrast yields very poor detection performance (AUC = 0.65) for this detection task. These results corroborate the 95% confidence intervals on difference in AUCs reported in the second column of Table 1.

**Table 1:** Comparison of OC-SVM and SPM classification performance. Data are differences in AUCs, with 95% confidence intervals in brackets. All differences are significant and in favour of OC-SVM, except for the detection of the blurred junction where no difference between the techniques can be shown.

	Blurred junction	heterotopy
OC-SVM vs SPM-junction	−7.622 × 10^−4^ [−2.2 × 10^−3^, 6 × 10^−4^]	0.2737 [0.2533, 0.2940]
OC-SVM vs SPM-extension	0.2888 [0.2835, 0.2951]	0.3478 [0.3445, 0.3507]
OC-SVM vs SPM-conjunction	0.1053 [0.1015, 0.1099]	0.2892 [0.2703, 0.3042]

The three coupled values of (TPR, FPR) are {(0.73, 0.01), (0.85, 0.05) and (0.88, 0.1)} for the OC-SVM and {(0.23, 0.01), (0.75, 0.05) and (0.87, 0.1)} for the SPM-junction classifier, thus underlining the higher performance of the OC-SVM classifier at low FPR which would be needed in clinical practice.

**Fig 8:**
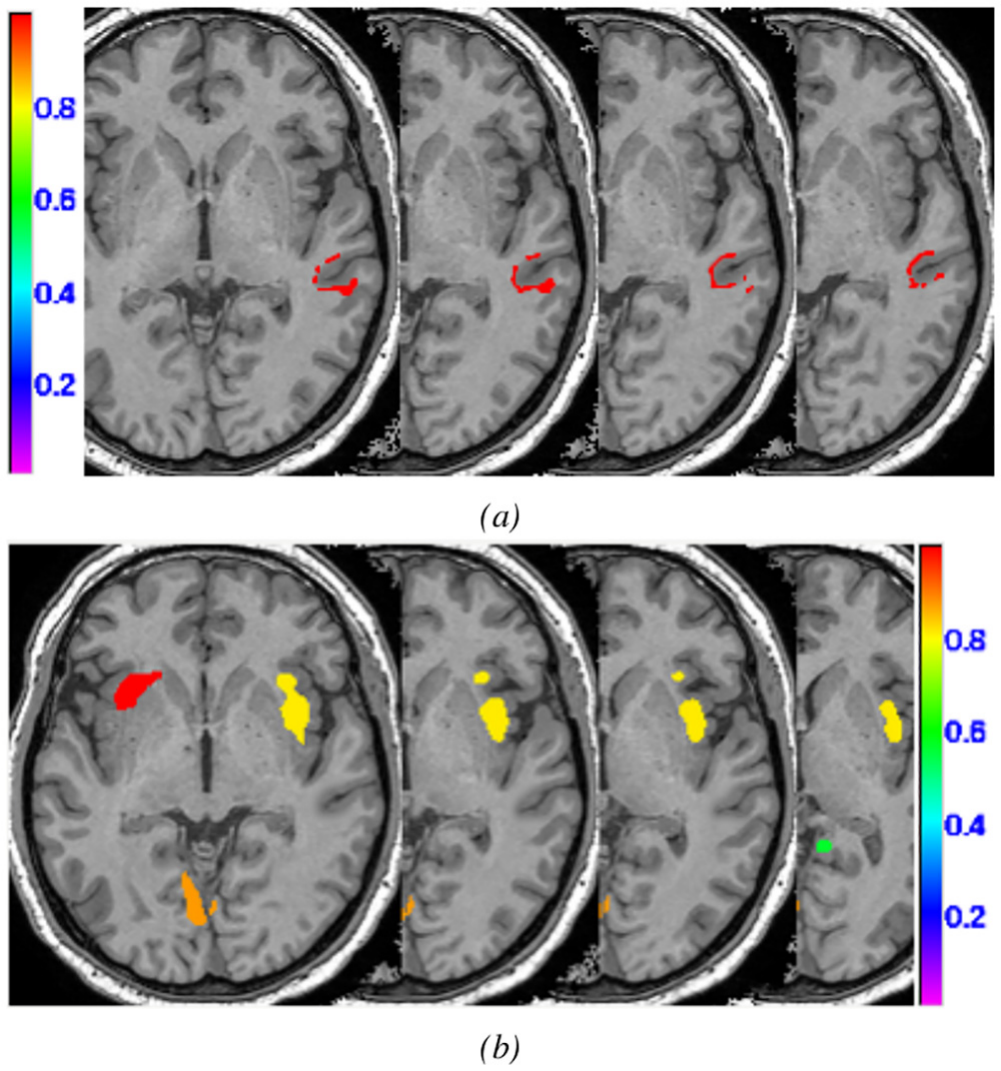
Example of OC-SVM and SPM labelled cluster maps for the blurred junction simulation.

Fig 8 gives an example of the detection maps obtained by OC-SVM and SPM analysis based on the junction map after thresholding the detection map at the same p-value of 0.001. This example illustrates that, for a reasonable false positive detection rate (0.1%), the SPM approach fails to retrieve the blurred junction lesion while the OC-SVM approach detects most of the lesion with high specificity. The ability of the OC-SVM to detect very subtle blurred junction lesions that are highly likely to be missed by standard visual inspection of the MR image is a very promising result.

For the heterotopion-like lesions, Fig 7b shows the ROC curves corresponding to the multivariate OC-SVM analysis as well as the SPM univariate analysis considering the junction and extension contrasts separately or the conjunction of both contrasts. The OC-SVM approach (AUC = 0.90) outperforms the SPM analyses based on the junction contrast (AUC = 0.62) and the conjunction analysis (AUC = 0.60) which both outperform the SPM analysis based on the extension contrast (AUC = 0.55) (see third column of Table 1). The ROC curve corresponding to the OC-SVM performance for heterotopion-like lesions in Fig 7b shows that the system was able to retrieve almost 70% of the global volume of the simulated lesions without any false positive detection. The coupled (TPR, FPR) values of the four methods were {(0.72, 0.01), (0.80, 0.05) and (0.83, 0.1)} for OC-SVM, {(0.09, 0.01), (0.28, 0.05) and (0.37, 0.1)} for SPM-junction, {(0, 0.01), (0.01, 0.05) and (0.03, 0.1)} for SPM-extension, and {(0, 0.01), (0.03, 0.05) and (0.07, 0.1)} for the conjunction of both SPM contrasts.

#### Clinical data results

Patients from each test group were tested using OC-SVM and SPM models estimated based on their respective normal control database. The OC-SVM distance map and all three SPM maps (based on the junction or extension contrasts or the conjunction of both contrasts) of each of the eleven patients were thresholded at the same p-value of 0.001. Table 2 summarizes the results obtained for the eleven patients with all four approaches.

**Table 2:** OC-SVM and SPM classification results for clinical data for a p-value of 0.001. The third column indicates the location of the epileptogenic lesions reported by the clinician for each patient. Columns 4 to 7 report the detection results of OC-SVM and the three SPM analyses. For each method, the number of false positive clusters is indicated in parentheses. The ^⋆^ in column 2 indicates the FCD lesions that were confirmed by histology.

Patient	Lesion	Location	OC-SVM	SPM junction	SPM extension	SPM Global null
#1 (MRI+)	#1^⋆^	precentral gyrus R	✓ (2)	✓ (3)	X (17)	X (1)
#2 (MRI+)	#2^⋆^	middle frontal gyrus L	✓ (3)	✓ (4)	✓ (34)	✓ (7)
#3 (MRI+)	#3	superior frontal gyrus R	✓ (0)	✓ (12)	✓ (24)	✓ (9)
#4 (MRI-)	#4 #5^⋆^	hippocampus R amygdala R	✓ (2) ✓	X (5) X	X (24) X	X (5) X
#5 (MRI-)	#6^⋆^	middle frontal gyrus L	✓ (1)	X (3)	X (3)	✓ (0)
#6 (MRI-)	#7 #8^⋆^	hippocampus R temporal R	X (7) ✓	X (11) X	X (13) ✓	X (5) ✓
#7 (MRI-)	#9	middle frontal gyrus L	X (3)	X (3)	X (9)	✓ (4)
#8 (MRI-)	#10	frontal L	✓ (10)	X (14)	✓ (56)	X (18)
#9 (MRI-)	#11	anterior temporal lobe R	✓ (4)	✓ (14)	✓ (3)	✓ (2)
#10 (MRI-)	#12	parieto occipital L	✓ (2)	✓ (5)	✓ (25)	✓ (14)
#11 (MRI-)	#13	temporal lobe L	X (1)	✓ (3)	X (23)	X (4)
**Sensitivity MRI+** **Mean FP MRI+**			3/3 1.7 FP	3/3 6.3 FP	2/3 25 FP	2/3 5.7 FP
**Sensitivity MRI-** **Mean FP MRI-**			7/10 3.7 FP	3/10 7.2 FP	4/10 19.5 FP	5/10 6.5 FP
**Overall Sensitivity** **Overall Mean FP**			10/13 3.2 FP	6/13 7.0 FP	6/13 21.0 FP	7/13 6.3 FP

**Fig 9:**
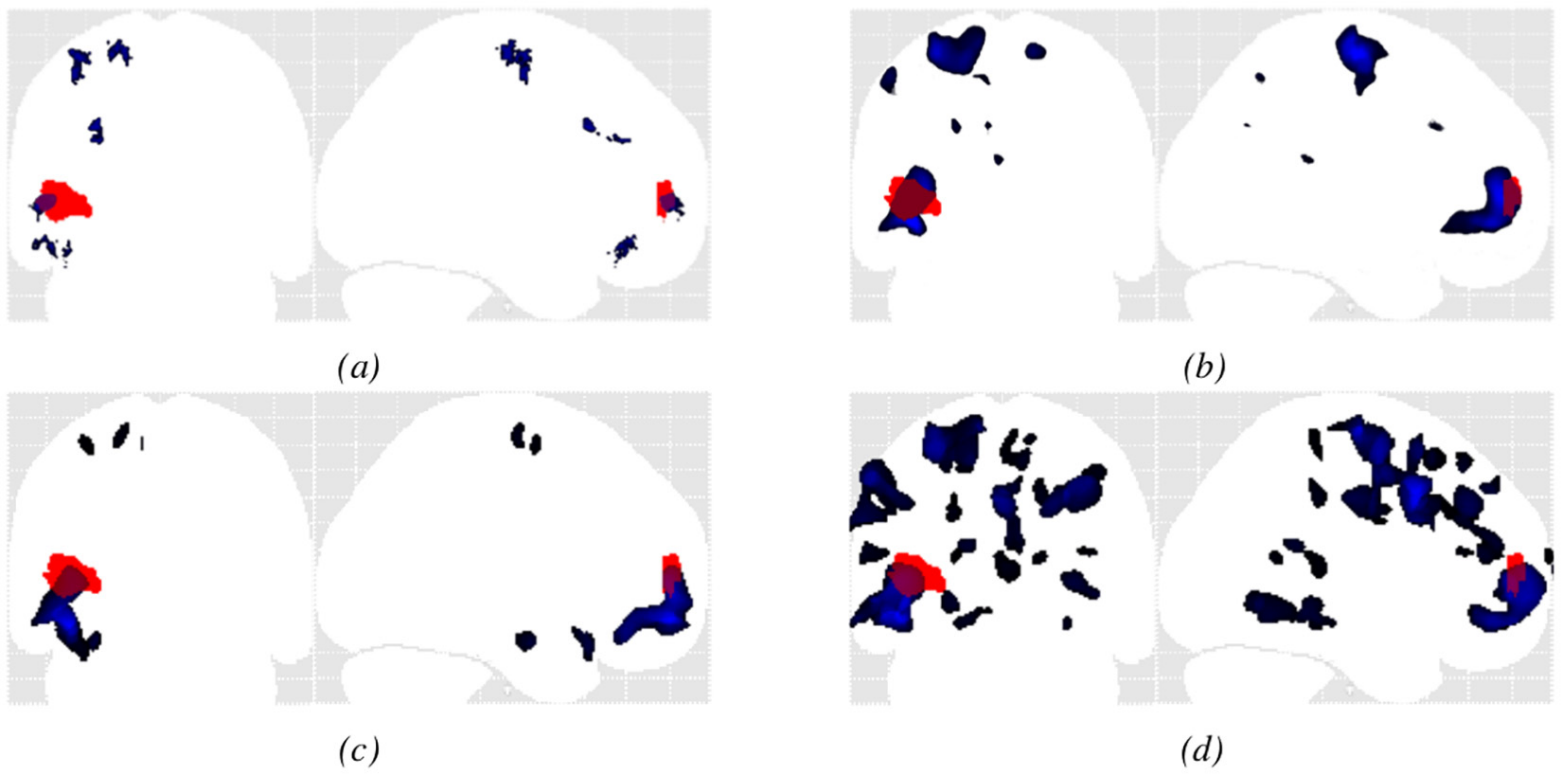
Example MIP of the detected cluster maps (blue) for patient #2 (MRI+) overlaid on the MIP of the expert delineated lesion (red). (a) OC-SVM distance map thresholded at *p* < 0.001; (b) SPM analysis based on the T-score map from the conjunction of both contrasts thresholded at *p* < 0.001 (c) SPM junction-based T-score map thresholded at *p* < 0.001; (d) SPM extension-based T-score map thresholded at *p* < 0.001.

*Results for MRI + patients (#1 to #3):* The OC-SVM approach succeeded in detecting 3/3 lesions reported by the neurologist, yielding a sensitivity of 100% for MRI+ lesions with an average of 1.7 FP detections per patient. The SPM junction based analysis achieved equivalent sensitivity with an average of 6.3 FP per patient. As for the simulation examples, the OC-SVM approach produced fewer false positive detections than all SPM analyses (>5.7 FP). Fig 9 compares the maximum intensity projection (MIP) of the classification results obtained by OC-SVM and SPM analyses based on the junction and extension map for patient #2 (see Table 2). Both methods succeeded in identifying the confirmed FCD lesion located in the left fronto-basal area. Comparison of cluster shapes and sizes in Fig 9a, 9b, 9c and 9d illustrates that the OC-SVM approach is more specific in lesion localization than the SPM approach. For this lesion resulting from a GM extension, the cluster detected by OC-SVM is indeed located exactly at the bottom of a sulcus, whereas the cluster detected by the extension based SPM analysis has a bigger extent and is smoother. The OC-SVM analysis produced 3 FP whereas the SPM analyses based on the junction and extension contrasts produced 4 FP and 34 FP respectively. The conjunction analysis of both contrasts produced 7 FP.

**Fig 10:**
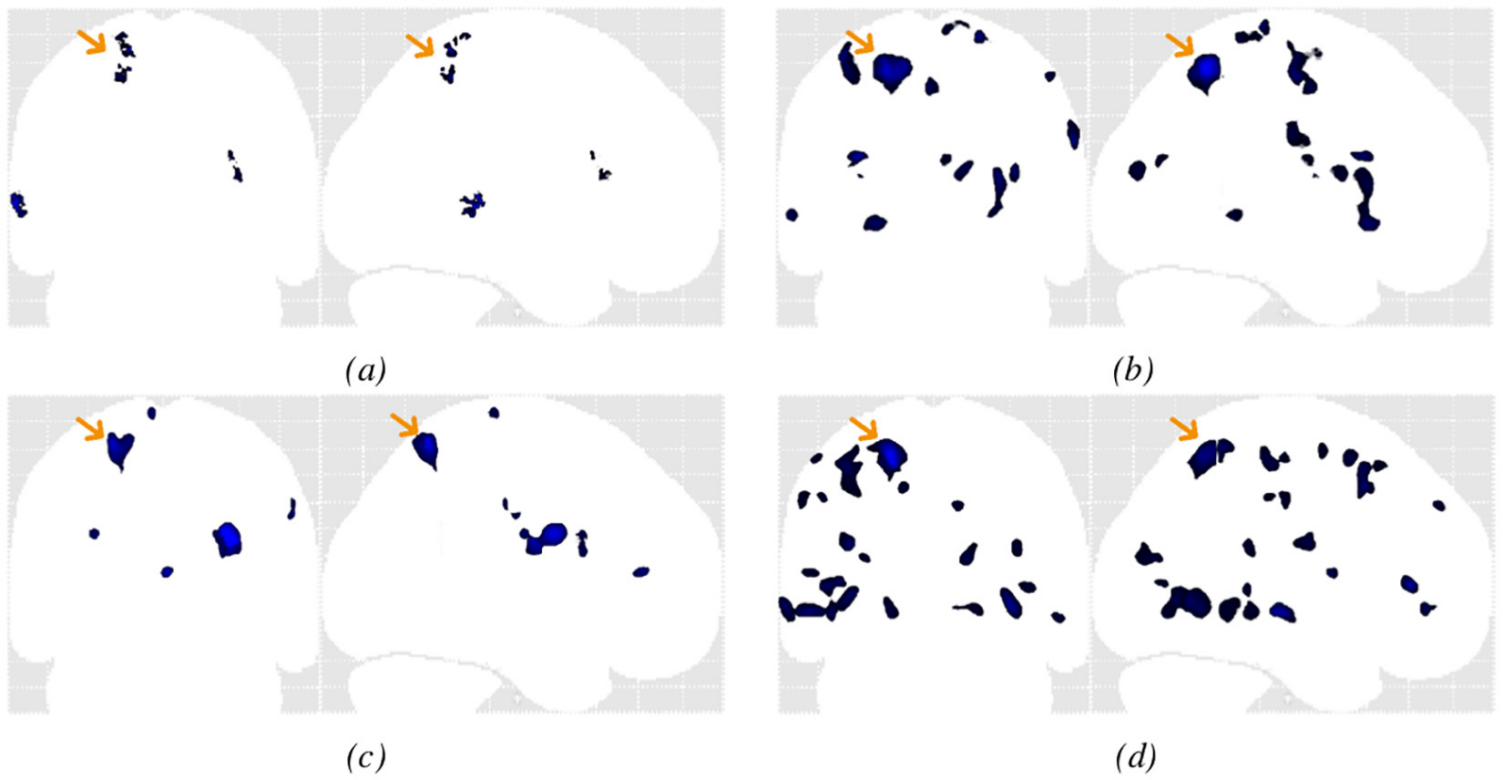
Example MIP of the detected cluster maps (blue) for patient #10 (MRI-), the presumed lesion is indicated with the yellow arrow. (a) OC-SVM distance map thresholded at *p* < 0.001; (b) SPM analysis based on the T-score map from the conjunction of both contrasts thresholded at *p* < 0.001 (c) SPM junction-based T-score map thresholded at *p* < 0.001; (d) SPM extension-based T-score map thresholded at *p* < 0.001.

*Results for MRI- patients (#4 to #11):* The OC-SVM approach succeeded in detecting 7/10 lesions, resulting in a sensitivity of 70% for MRI- lesions with an average of 3.7 FP detections per patient. It missed the lesions in patients #7 and #11. Two clusters were correctly detected at these two lesion locations from the thresholded OC-SVM score map but they were discarded because of their small size (size <82 voxels). When selecting a higher p-value of 0.005, these two lesions are well detected at the price of a decreased specificity (13 FP for patient #7 and 15 FP for patient #11 against 3 FP and 1 FP for p = 0.001, data not shown). Among all SPM analyses, the conjunction of both contrasts achieved the best detection performance in terms of sensitivity and the number of false positive detections, by detecting 5/10 lesions (50% sensitivity) with an average of 5.7 FP detections per patient. The conjunction of both contrasts allowed detecting the lesion in patient #7 that was initially missed by both individual contrasts. All SPM analyses missed the lesion in patient #4 located in the right amygdala. Fig 10 compares MIPs obtained for patient #10. The lesion for this patient was not spotted after visual inspection of the MRI. The other exams including SEEG, VEEG as well as FDG-PET and MEG, however, all colocalized the presumed lesion in the left parietal lobe. Fig 10a, 10b, 10c and 10d illustrate the higher specificity obtained using the OC-SVM approach.

*Overall performance:* Considering all eleven patients, the OC-SVM approach detected 10/13 lesions (77% overall sensitivity) with an average of 3.2 FP. The SPM conjunction analysis detected 7/13 lesions (54%) with an average of 6.3 FP. Both SPM analyses based on individual contrasts detected 6/13 lesions (46%) with an average of 7 FP for the junction contrast and 21 FP for the extension contrast. All approaches considered allowed a better sensitivity than the visual inspection of the T1-weighted MR scans that only allowed the detection of 5/13 lesions (39% sensitivity).

### Computation time

All results were obtained on a grid featuring 14 Intel Xeon quad-core L5420 2.50 GHz with 16 GB RAM memory. The main workload was associated with learning the 1.5 million OC-SVM models. If treated in sequential mode, this learning time was ≈ 28 hours while the feature extraction step and the score map computation for any test patient took about 12 and 15 min respectively. We therefore developed a parallel implementation based on the octree method [42]. This method allows partitioning a three dimensional space by recursively subdividing it into eight octants. The final number of octants was set as to get a fixed maximum number of voxels in each octant. Each octant was then processed as an independent thread. In accordance with our grid specifications, each octant was composed of about 35000 to 60000 voxels. To process the whole 3D volume, 30 threads were executed in parallel on the grid. This allowed reducing the model learning time to less than 20 min and the two other processing times (feature extraction and score map computation) to less than 5 min.

## Discussion

We designed and implemented an automated voxelwise detection system of common epileptogenic lesions in MR brain imaging based on machine learning of specific features of these lesions. The main contribution is 1) to consider this diagnostic task as a novelty detection problem that is solved using the OC-SVM methodology and 2) to perform a multivariate voxel-based analysis yielding a cluster map quantifying the probability of a cluster of being abnormal. Performance of the multivariate OC-SVM based system was compared with that of the reference mass univariate SPM method. This comparison was first performed using simulated MR data mimicking standard epileptogenic lesions, and then using eleven patients, including 3 MRI+ and 8 MRI- exams.

The choice to perform a voxel-based analysis was motivated by the clinical need to develop an automated diagnostic system that can assist clinicians to non-invasively and accurately detect epileptogenic lesions of varying size anywhere on MRIs of the brain. Clinically, the decision for surgery and/or electrode placement is not solely based on imaging; detecting an abnormality as a target for further exploration is very important especially in MRI-negative patients [43]. The intuitive way to address this question would be to consider a standard binary classification problem. As underlined in the introduction, this however raises the issue of learning with imbalanced data [41, 44] and collecting enough samples from the pathological class to accurately model the intra-class variability. These led us to consider this challenging task as a novelty detection problem, which consists in constructing the predictive model from normal training samples only. Our choice to define one predictive OC-SVM model for each of the 1.5 millions voxels was aimed at accounting for the tissue and region specific feature distribution mentioned above. It was also inspired by the standard mass univariate statistical analysis developed in the neuroimaging community over the past few years. Our hypothesis was that the OC-SVM analysis could outperform the standard SPM analysis by enabling multivariate instead of mass univariate analysis.

Another novel contribution of this study is the method that we propose for converting the OC-SVM scores for a given test image into probabilities. This relies on the observation that typical epileptogenic lesions are very small in comparison with the entire volume of interest (less than 1%) and that we obtained similar distributions in terms of shape and skewness for the OC-SVM scores for patients and for healthy controls (see example in Fig 4). This method that considers pathological samples as outliers of the normal score distribution may also be suitable for other pathologies based on subtle variations of the normal pattern.

The proposed system was cross validated with a SPM analysis based on junction and extension maps.

For the simulation data, the CAD system based on the OC-SVM had an overall higher AUC than the SPM analysis for the two kinds of simulated lesions (heterotopion and blurred junction) and a higher sensitivity at very low FPR. For blurred-junction like lesions, unlike the SPM analysis, the OC-SVM approach was able to detect, with both high specificity and sensitivity, very subtle lesions that are very likely to be missed by standard visual inspection of the MRI (see Fig 8).

For the clinical data, the proposed approach successfully detected 100% of the lesions in the MRI positive patients with less than 2 FP per scan while the SPM analysis based on the junction contrast achieved similar sensitivity at the price of about four times more FP per scan. For the MRI negative cases, the OC-SVM based approach outperformed all SPM analyses by combining both highest sensitivity (70%) and highest specificity (less than 4 FP per scan).

Performance achieved by OC-SVM compares well with the-state-of-the-art achieved in the recent study by Hong et al. [20] that presented an automated algorithm for the detection of FCD type II in MRI- patients based on a two step classification scheme. The first step combined a linear discriminant analysis (LDA) with six surface based features (including cortical thickness) derived from the extraction of the inner and outer cortical surface from T1-weighted MR images. The resulting series of detected clusters (connected vertices) including true positive and false positive clusters (about 30 false positives per patient) were then passed through a second cluster-based LDA to remove the residual false detections. Results reported in their study showed a high sensitivity (≈14 detected lesions out of 19 annotated lesions) and a good specificity with an average of 1-3 false positive detections per patient. These results are comparable with those achieved by our OC-SVM system in a one step procedure which successfully detected 7/10 MRI- lesions in eight patients with an average of 3-4 false positive detections per patient. In our study, unlike in Hong et al. [20], we did not evaluate the specificity of the proposed system in healthy control subjects. However, we can deduce the achievable performance from the simulation study. For all simulation subjects, the proposed system identified the simulated lesion with no detections outside the simulated lesion location which suggests high specificity of our system in healthy controls. In another recent study, Ahmed et al. [21] also proposed to combine five surface-based measures of cortical thickness at the vertex level with an ensemble classifier consisting of bags of 10 base-level classifiers trained using logistic regression. The authors evaluated the performance of this CAD configuration on 31 patients with FCD (7 MRI+ and 24 MRI- scans). Their approach detected 86% of the FCD lesions in the MRI+ group and 58% of the FCD lesions in the MRI- group. The author’s approach was more sensitive than a mass univariate statistical analysis (SPM) based on the cortical thickness feature alone, but had a lower specificity.

The drawback to considering a voxel-wise classification scheme is that it requires registering all subject’s brain images into a common space. In this study, we used the unified segmentation algorithm [29] to register all subject’s images to the MNI space. A recent study by Klein et al. [45] compared several registration algorithms of healthy control subjects brain images. In this comparison, the DARTEL method that was introduced by Ashburner in 2007 [46] as an alternative to the unified segmentation algorithm was found to be more accurate. In our present study, we tested both registration methods on our data. No significant gain in performance was achieved by using DARTEL instead of the unified segmentation approach (data not shown).

It is difficult to establish a fair comparison between the performance achieved by the SPM analyses in our study and those published over the last few years [16, 18, 19] because of the heterogeneity of the patient populations, and annotation and evaluation protocols. Thesen et al. [19], for instance, performed an SPM analysis using surface-based features, cortical thickness and GM/WM contrast. The best performance was found for the cortical thickness and GM/WM contrast. Further comparison of the ROC curve analysis, however, is difficult because Thesen et al. used a definition of TP (a patient with one detected cluster in the lesion area) and FP (healthy control subject with detected clusters) different from that defined in the present study. While we used a fixed Gaussian kernel width of 6 mm to smooth the feature maps, it is unlikely that this parameter is important in the context, as only a marginal influence was found in [19] for typically used values between 5 and 12 mm. Results of the SPM analyses in our study are also in accordance with those obtained by Bruggemann et al. [18] showing that conjunction analyses based on GM and WM maps allowed significant performance improvement as compared with the SPM statistical maps derived from a single contrast (WM or GM). It should be noted that the OC-SVM method presented here can easily be extended to any number of additional features.

One drawback of the novelty detection algorithm is that the model does not learn specific patterns of the lesions. We tried to alleviate this limitation by incorporating only features that were previously reported to be discriminant in the task of identifying FCD lesions. Wagner et al. [32], for instance, demonstrated a 29% sensitivity gain induced by the use of MRI based junction and extension feature maps to visually detect FCD type IIa lesions in addition to the conventional MR T1-weighted images.

In the present study, we hypothesized that these two features will allow discriminating malformative lesions including FCD and heterotopia from normal brain tissue; choosing just two feature maps also allowed an easy comparison with SPM. The OC-SVM framework, however, offers a great flexibility in adding other types of features such as surface-based measures of cortical thickness investigated in recent studies [20, 21]. An improvement in performance is however not necessarily guaranteed as it depends heavily on the correlation between the added features and the target lesions and on the number of available training samples. In our preliminary study [28], we evaluated a CAD scheme based on learning a OC-SVM model with six features including the junction and the extension features as well as the cortical thickness and the three tissue probabilities for the GM, the WM and the CSF. No loss in classification performance was observed in the present study.

For the clinical data, we set the threshold on the size of the detected clusters to 82 voxels for both OC-SVM and SPM analyses. FCD and nodular heterotopia lesions usually have a size of 1 to 2 cm in diameter. In terms of voxels of the size used in this study, this would correspond to a volume of 120 to 5000 voxels. Considering only the clusters over 82 voxels would therefore in general not result in missing typically sized FCD or heterotopia lesions. A lower threshold would likely produce small false positive detections that would then have to be discarded by an expert when inspecting the cluster map outputted by the CAD system visually. We believe the current thresholds are a reasonable compromise, but if the system is widely taken up further refinements are entirely envisageable.

A future direction is to incorporate different types of spatial priors in the learning steps and also to investigate the possibility of going towards a multi-modal CAD system for intractable epilepsy detection by incorporating additional features extracted from other imaging modalities such as the FLAIR sequence in MRI or FDG-PET scans. While the CAD system developed in this study was designed to detect epileptogenic lesions, the framework depicted in Fig 2 is in principle suitable for detecting other pathologies characterized by small lesions on brain MRI. Examples would include the detection of plaques in multiple sclerosis, vascular white matter lesions in normal ageing and as a risk factor for stroke, focal atrophy in dementia, etc. The feature selection and extraction steps can be adjusted to the specific pathology. Importantly, the OC-SVM outlier detection step and the method to control false positives developed in this paper could be used for such applications.
